# Bacteriophage T4 Infection of Stationary Phase *E. coli*: Life after Log from a Phage Perspective

**DOI:** 10.3389/fmicb.2016.01391

**Published:** 2016-09-08

**Authors:** Daniel Bryan, Ayman El-Shibiny, Zack Hobbs, Jillian Porter, Elizabeth M. Kutter

**Affiliations:** ^1^Kutter Bacteriophage Lab, The Evergreen State CollegeOlympia, WA, USA; ^2^Biomedical Sciences, University of Science and Technology, Zewail City of Science and TechnologyGiza, Egypt; ^3^Faculty of Environmental Agricultural Sciences, Arish UniversityArish, Egypt

**Keywords:** bacteriophage T4, *E. coli*, hibernation mode, multiplicity of infection (MOI), scavenger response, stationary phase, sigma S, T4 nucleotide synthesizing complex

## Abstract

Virtually all studies of phage infections investigate bacteria growing exponentially in rich media. In nature, however, phages largely encounter non-growing cells. Bacteria entering stationary phase often activate well-studied stress defense mechanisms that drastically alter the cell, facilitating its long-term survival. An understanding of phage-host interactions in such conditions is of major importance from both an ecological and therapeutic standpoint. Here, we show that bacteriophage T4 can efficiently bind to, infect and kill *E. coli* in stationary phase, both in the presence and absence of a functional stationary-phase sigma factor, and explore the response of T4-infected stationary phase cells to the addition of fresh nutrients 5 or 24 h after that infection. An unexpected new mode of response has been identified. “Hibernation” mode is a persistent but reversible dormant state in which the infected cells make at least some phage enzymes, but halt phage development until appropriate nutrients become available before producing phage particles. Our evidence indicates that the block in hibernation mode occurs after the middle-mode stage of phage development; host DNA breakdown and the incorporation of the released nucleotides into phage DNA indicate that the enzymes of the nucleotide synthesizing complex, under middle-mode control, have been made and assembled into a functional state. Once fresh glucose and amino acids become available, the standard lytic infection process rapidly resumes and concentrations of up to 10^11^ progeny phage (an average of about 40 phage per initially present cell) are produced. All evidence is consistent with the hibernation-mode control point lying between middle mode and late mode T4 gene expression. We have also observed a “scavenger” response, where the infecting phage takes advantage of whatever few nutrients are available to produce small quantities of progeny within 2 to 5 h after infection. The scavenger response seems able to produce no more than an average of one phage per originally available cell, and few if any further progeny are produced by cells in this mode even if fresh nutrients are made available later.

## Introduction

Bacteriophage infection has traditionally been studied using bacterial hosts growing exponentially, with active aeration, in one of a few well-studied media. The resurgence of interest in therapeutic, prophylactic, and agricultural phage applications, as well as growing awareness of the substantial environmental impact of phages, make it increasingly important to study the details of phage-host interactions under conditions that are more similar to those encountered in natural environments. Such exploration is especially important since the emergence of multi-drug resistant pathogenic bacteria has become a major public health concern (World Health Organization [WHO], 2014). Understanding how cellular changes affect phage infection under natural conditions is essential for the success of the many proposed antimicrobial phage applications. We here explore the ability of coliphage T4 to infect hosts that are in stationary phase. Such studies are particularly relevant as close relatives of this carefully studied model organism are ubiquitous in nature and have been very widely used in cocktails for treating enteric infections (Brüssow, 2005; Sarker et al., 2012; Kutter et al., 2014; Sarker et al., 2016).

T4 infection of *E. coli* has been one of the most thoroughly studied model systems in molecular biology and microbiology for over 60 years (Mathews et al., 1983; Karam et al., 1994; Miller et al., 2003). T4 infection of exponentially growing *E. coli* quickly disrupts host genome structure and expression, largely by making use of T4’s complete substitution of HMdC for dC in its DNA (Kutter et al., 1994c). Transcription of all cytosine-containing DNA is blocked, translation of residual host RNA is universally cut off, host DNA is gradually degraded, and the infected cell cannot respond to a change in available carbon sources. However, it was widely believed that T4 does not multiply in stationary phase cells, since T4 plaques don’t continue to grow in size after the lawn is fully formed (Benzer, 1952; Adams, 1959). Schrader et al. (1997a,b) reported that T4 was incapable of multiplying in *E. coli* AB1157 that had been starved for 24 h in Lysogeny Broth (LB), even though 94% of the T4 phage particles bound to the host. They found, in contrast, that both coliphage T7 and *P. aeruginosa* phage UT1 infected their hosts successfully in identical conditions, though with extended latent periods and reduced burst sizes; plaques of T7 on a plate will also continue to grow in size indefinitely (Yin, 1991). Other indications have been found that T4 may multiply less efficiently than T7 in the gut environment. For example, in axenic mice monocolonized with *E. coli*, the amount of T4 was seen to increase only 300-fold during transit of the gut as compared to passive transit in control axenic mice, while T7 showed a 10^6^ fold increase (Weiss et al., 2009).

Optimal infection strategies might well be quite different when T4 tries to infect stationary-phase cells – as must frequently happen in the mammalian colon, *E. coli*’s primary habitat, where the microbial density is extremely high and competition for nutrients is intense. Whereas σ^70^ is the main regulator for “housekeeping” gene transcription in exponential-phase enteric bacteria, σ^S^ (regulated by gene RpoS) plays a central role for cell adaptation during stationary phase. There, *E. coli* maintains a basal metabolic level that lets it scavenge for nutrients from dead cells and respond quickly to a wide range of new nutrients (Hengge-Aronis, 1993; Zambrano and Kolter, 1996). Many new pathways are activated as the overall metabolic rate decreases during the transition to stationary phase, largely under the control of σ^S^. *E. coli* in stationary phase have denser cell membranes, with altered phospholipids and expanded periplasmic spacing. They pack their chromosome more tightly and produce a range of protective enzymes such as catalase (Huisman et al., 1996; Págan and Mackey, 2000). The cells maintain a low level of synthesis of some proteins such as various transporters throughout stationary phase, enabling them to respond to new nutrients and to quickly resume vegetative growth (Huisman et al., 1996). The number of ribosomes is reduced (Cangelosi and Brabant, 1997), and many of those remaining are dimerized to a storage format, to be rapidly released when nutrients are supplied (Wada et al., 2000).

Here, we describe in substantial detail the patterns of the interaction when T4 infects *E. coli* 48 h after inoculation into fresh minimal medium, identifying a new “hibernation” mode of long-term interaction there, and explore the ability of T4 to produce infective centers in *E. coli* that are up to several weeks into stationary phase.

## Materials and Methods

### Media

Stationary phase studies were conducted in M9 minimal medium [25 mL 20X M9 salts (120 g Na_2_HPO_4_⋅H_2_O, 60 g KH_2_PO_4_ (anhydrous), 10 g NaCl, and 20 g NH_4_Cl per 790 mL DIH_2_O), 0.5 mL 0.1 M FeCl_3_, 5 mL, 0.1 M MgSO_4_, 0.5 mL 0.1 M CaCl_2_, per 461 mL DI H_2_O, sterile filtered and supplemented with 5 mL casamino acids (CAA; 10% w/v) and 3 mL glucose (20% w/v)]. Tryptic soy broth (30 g tryptic soy broth powder per 1000 mL of DI H_2_O) was used for standard cultivation of bacteria and propagation of phage. Phage buffer (10 mL 1 M Tris pH 7.6, 0.1 g gelatin, 4 g NaCl, 990 mL DI H_2_O, boiled and pH balanced to 7.6 at 25°C, autoclaved) was used for phage stocks and as a diluent when titering phage.

### Bacterial Strains

Studies were conducted in *E. coli* W3110 derivatives ZK126 (Δ*lacZ rpoS*+), and ZK1000 (Δ*lacZ rpoS*-) (the kind gift of Dr. Steve Finkel) and phage were routinely plated on ZK126. Amber suppressor strain CR63 from the Evergreen collection was also used. Stocks were maintained in 20% (v/v) glycerol at -80°C. All cultures were grown and infections carried out in Erlenmeyer flasks in a gyratory water bath at 37°C and 120 RPM.

### Phage Strains and Their Propagation

Phage T4D was from the Evergreen collection, as was T4 gene 43 (DNA polymerase) *amber* mutant am4332. T4 was propagated in ZK126 while am4332 was propagated in amber suppressor *E. coli* strain CR63. Briefly, a 500 mL culture at ~0.4 OD_600nm_ (OD, all optical density readings used throughout are done at 600 nm) in TSB was infected at a multiplicity of infection (MOI) of ~0.1, then incubated at 120 RPM and 37°C for ~4 h before lysing with 1–2 mL of CHCl_3_. The lysates were incubated statically at room temperature overnight to allow endogenous nucleases to degrade host DNA and then centrifuged for 10 min at 4642 × *g* to pellet bacterial debris. The supernatant was decanted away from the pellet, spun at low speed a second time, and then centrifuged for 2 h at 23,975 × *g* to obtain a concentrated phage pellet. This was resuspended without agitation in a few mL of phage buffer overnight at 4°C.

### Infection of *E. coli* in Stationary Phase

**FIGURE 1:**
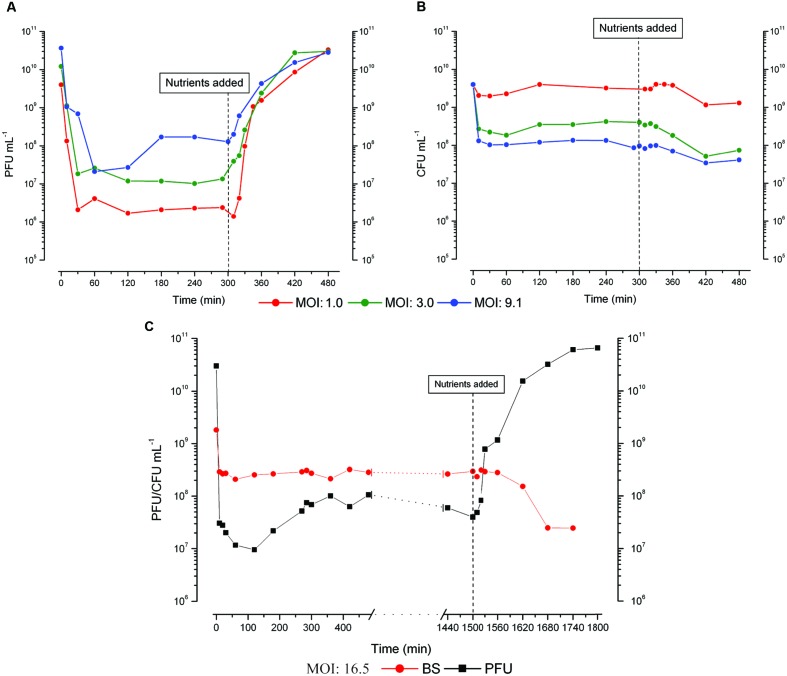
**Representative figures of (A) phage production and (B) bacterial survivors (BS) when the infection was carried out at three different multiplicity of infection (MOI) in parallel flasks split from the same 48 h old culture of *E. coli* ZK126, with 0.12% w/v glucose and 0.1% w/v CAA re-added 5 h after infection (As described in Section “Materials and Methods,” chloroform is always added to the phage samples, so these values include all complete phage, both intracellular and free). (C)** Bacterial killing and phage production when ZK126 was infected after 48 h with T4 at an MOI of 16.5 and no further nutrients were added until 25 h after infection. Both a long hibernation mode and a small scavenger response are observed. Supplemental Material with further representative figures showing similar behavior are provided for **Figure 1**, **Figure 2**, and **Figure 3A** as well as for the response of uninfected cells to nutrient addition.

**FIGURE 2:**
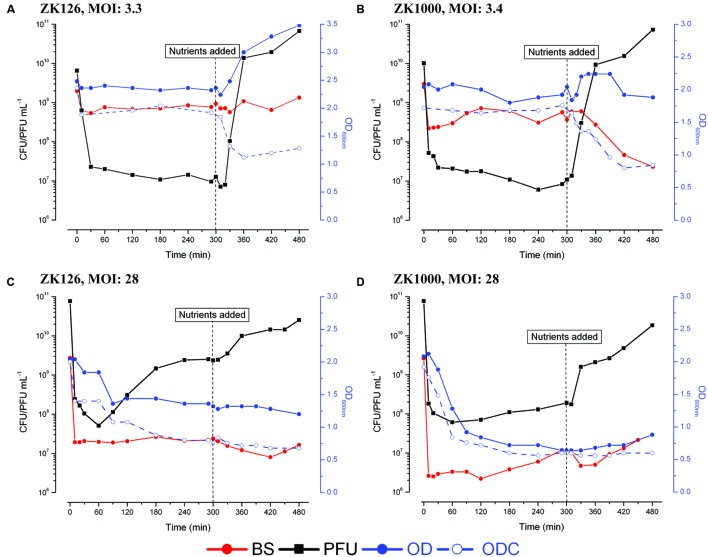
**Representative figures of phage production, bacterial killing, and optical density without (OD) and with (ODC) the addition of chloroform.** 0.12% w/v glucose and 0.1% w/v CAA added 5 hr after infection. **(A)** ZK126, MOI: 3.3, **(B)** ZK1000, MOI: 3.4, **(C)** ZK126, MOI: 28, and **(D)** ZK1000, MOI: 28.

Overnight cultures used for stationary phase infections or for phage plating were grown either from 3 to 5 colonies taken from plate cultures no more than 3 days old or directly from a scraping of a glycerol freezer stock stored at -80°C. This latter, less common method is used routinely in the Finkel and Kolter labs for stationary phase work, and usually gives less variable bacterial behavior in stationary phase cultures than does picking individual colonies. We adopted this technique in the fall of 2014 and the experiments presented in **Figures 1** and **2** were inoculated by this method. Stationary phase infections were performed with cultures of *E. coli* ZK126 or ZK1000 in M9 at a range of multiplicities of infection (MOI), as indicated. Either 5 or 24 h post-infection, nutrients were added to bring the concentration in the infection flask up to 0.12% w/v glucose and 0.1% w/v casamino acids. The infected culture was tracked for an additional 3–5 h after nutrient addition (representative figures showing the nutrient response of uninfected cells are provided as Supplemental Material).

Phage were tracked as plaque forming units (PFU), enumerated by taking a 30 μL sample and adding it to a microcentrifuge Eppendorf tube containing 270 μL of phage buffer and 30 μL of chloroform. The samples were shaken well and allowed to settle for at least 20 min before 30 μL of the sample was serially diluted through 96 well plates containing 270 μL of phage buffer. Then 100 μL of each appropriate dilution was added to a tube containing 3 mL of molten top agar held at 45°C and 100 μL of ZK126 at an OD of about 0.5, gently mixed well and poured onto a TSA plate. Bacterial survivors (BS) were determined by taking 30 μL samples, serial diluting through 96 well plates containing 270 μL M9 and spread plating 100 μL aseptically on round TSA plates. Optical density was determined after diluting samples either two or fourfold in M9 as needed to bring the OD below 1.0.

Duplicate samples collected at the same times had ~100 μL of chloroform added to the dilution medium. These samples were shaken and left at least 20 min to allow the chloroform to settle; then the OD of a 600 μL aliquot was read. (Chloroform is used to indicate successful production of lysozyme, indicating some phage early gene expression. When exposed to chloroform, phage-infected cells that have already produced some lysozyme will lyse, while uninfected cells will die but generally remain structurally intact Adams, 1959).

### Host DNA Degradation Experiments

To explore how stationary-phase phage infection affects the host DNA, cultures of exponential-phase ZK126 were labeled with tritiated deoxythymidine (^3^H-dT). At OD = 0.4 on the first day, 5 mL were transferred to flasks containing 0.25 mL dA (2 mg/mL in H_2_O) and 0.25 mL dT (100 μg/mL dT with 10 μCi/mL methyl ^3^H-dT) and incubated at 37°C in a shaking water bath while the remainder was grown in parallel without label to enumerate CFUs and PFUs and check the response to nutrient addition. At 48 h, both radio-labeled and unlabeled cultures were infected with either T4D^+^ or T4 am4332 at an MOI of about 10 phage per cell. The standard nutrient mixture was re-added to the unlabeled flasks at 5 h after infection (0.12% w/v glucose and 0.1% w/v casamino acids) to follow the ability of these infected cells to respond to nutrients by producing phage.

Labeled DNA samples were collected by spotting 50 μL samples onto filter paper disks and letting them dry for one min, placing them into a bath of 10% cold acetic acid for at least 10 min, washing them twice with cold 5% acetic acid for 10 min, then with 90% ethanol for 10 min and drying them. Samples were counted in a Packard 2200CA Tri-Carb Liquid Scintillation Counter using Perkin-Elmer Cybergold as the fluor.

[Note that *E. coli* can incorporate external deoxythymidine (dT) into its DNA only in the presence of 100 mg mL^-1^ deoxyadenosine (dA), to compete for a resident glycosidase (Boyce and Setlow, 1962)]. At least in exponential phase, T4 reuses released dT so efficiently for its own DNA synthesis that the breakdown can be observed only by using a phage mutant unable to make DNA, even when orders of magnitude more unlabeled dT is added to the culture (Kutter and Wiberg, 1968). Therefore, T4 DNA polymerase mutant am4332 was used for these studies in parallel with wild-type T4 to follow host DNA degradation in the absence of potential reincorporation of labeled host DNA into progeny phage.

## Results

### Patterns of T4 Infection of *E. coli* in Stationary Phase:

To explore T4 infection of starved *E. coli*, a 48 h old culture of ZK126 grown in M9 supplemented with glucose and CAA was infected at a range of different MOIs. By 5–10 min post-infection, more than 90% phage binding and MOI-dependent bacterial killing was routinely observed in all conditions. The pattern of phage production is consistently different between when there are only a few infecting phage per cell and when the MOI is much higher (cf. **Figure 1A**). At lower MOIs, no phage are produced until fresh nutrients are provided, but the infected cells respond and produce phage very rapidly when glucose and CAA are re-added. This phage production is seen so quickly as to suggest that early stages of phage infection had already been initiated, rapidly yielding a substantial burst of progeny phage. We refer to this behavior as “hibernation” mode.

In contrast, with high MOIs, we also observe what we call a “scavenger” response, where some phage production begins within a couple of hours after the phage are added to the cells without any addition of fresh nutrients, seldom extending beyond 4 h after infection and showing little if any ability to produce more phage when nutrients are re-added either 5 or 24 h after phage infection, presumably due to the fact that the phage infection process has been completed.

The behavior of T4 during the infection process appears to be determined on a cell by cell basis, and at least two different sorts of responses can be observed in a single infected culture, as seen in the MOI 9 infection in **Figures 1A,B** and the MOI 16.5 infection in **Figure 1C**. There, we observed an increase in phage titer before nutrient addition, but each infected culture still rapidly produced large quantities of progeny phage after nutrient addition. Those infected stationary phase cells that enter hibernation mode are still able to respond with a similarly large burst of phage (about 200 phage per initial cell over 4 h) if the nutrients are not added until 25 h after infection (**Figure 1C**). The drop in bacterial titer starting by 2 h post nutrient addition indicates that some of the phage-producing cells have been lysing, releasing phage that can now kill any still-uninfected phage-sensitive cells.

While patterns of phage production remained quite consistent from experiment to experiment, bacterial parameters such as the titer at 48 h, extent of killing after infection at a particular MOI, and grow-back of BS after infection varied between experiments. However, bacterial parameters in infections started in parallel from the same overnight culture remained very consistent. We hypothesize that the variation is due to differences in the initial bacterial cells in the cultures, some of which may have then developed into faster growing cells such as Growth Advantage in Stationary Phase (GASP) mutants or something that could more easily be killed etc. during the periods of stationary-phase incubation between the inoculation of the overnight culture from a freezer stock and infection of the experimental culture 72 h later (Zambrano and Kolter, 1996).

### Further Characterization of Scavenger versus Hibernation Modes

Following the optical density of each culture with and without adding chloroform to the sample (ODC and OD) provides a basis for better understanding what is happening in each of the two observed modes of infection of stationary phase cells. A drop in the OD very shortly after phage addition is probably indicative of “lysis from without” due to damage caused by the infection process (Abedon, 2011). A drop in the ODC indicates that at least some lysozyme has already been produced in the cell. The T4 lysozyme gene is transcribed at low levels from an early promoter 2.9 kb away (69.9 kb on the genetic map) with an immediately adjacent strong late promoter directing much higher levels later at 67 kb on the genetic map (Kutter et al., 1994b), but the lysozyme can’t reach the peptidoglycan layer as long as the inner cell membrane is intact. Exposing infected cells to chloroform induces lysis if any lysozyme has already been produced, while most uninfected cells remain unlysed even though they are killed (Adams, 1959). Thus, by following the susceptibility of the culture to lysis by chloroform, one can quickly determine whether early phage proteins are indeed being made. To explore the role of the stationary-phase sigma factor in determining response to phage, we here also include data on infection of ZK1000, isogenic except that RpoS has been deleted.

After infection of either host at fairly low MOI, which is what would most likely occur in nature, the OD and ODC may initially drop, as does the BS, but they then are relatively stable until nutrients are added (**Figures 2A,B**). The rapid, large production of phage seen after addition of nutrients is accompanied by a substantial increase in OD, while the ODC and number of BS decrease markedly. Since the cells are not dividing, the increase in OD is probably due to gradual enlargement of each cell, as occurs in exponential-phase T4 infections (presumably to accommodate the high phage production), and phage are then being released that can kill the bacteria that survived the initial infection. (Note that the number of unadsorbed phage prior to nutrient addition is far too low to account for this killing.)

In the case of the high MOI infections (**Figures 2C,D**), where a scavenger response is more frequently observed, we saw a drop in OD in both strains fairly soon after infection. In ZK126, the OD without chloroform only fell 8% by 30 min. After 60 min gradual phage production was observed for the next 3 h, reaching an average of ~1 phage per initial cell. By 90 min, there is an OD drop of 32%, presumably reflecting some cell lysis and release of the newly made phage. There is 46% lysis observed quite early in the presence of chloroform, implying that some lysozyme has been produced in those cells; the ODC falls an additional 16% before nutrient addition, while the OD remains relatively stable. BS titers as enumerated by CFUs remain stable until the addition of nutrients, suggesting that few free phage are present.

Shortly after infection of ZK1000, substantial lysis both with and without chloroform is observed, which may represent lysis from without and/or higher susceptibility to the early lysozyme being produced internally, something to which ZK1000, which lacks the protective adaptations of σ^S^, may be more sensitive. This lysis would release some nutrients to the remaining infected and uninfected cells. Still, relatively little phage production is seen until after the addition of nutrients at 5 h (**Figure 2D**). In ZK126, most of the pre-nutrient addition OD drop seen without chloroform addition occurs much later, after phage production begins, and the majority of phage production occurs 2 to 4 h after infection. In summary, when infecting stationary phase cells T4 is capable of at least two quite different patterns of infection. T4 can appear to lie relatively dormant in the cell, in what we call “hibernation” mode. Here, it initiates the infection process but produces no progeny phage until appropriate fresh nutrients are added, whereupon it rapidly makes large numbers of phage; relatively few differences are seen whether the nutrients are added at 5 or 25 h after the initial infection (**Figures 1A–C**). Levels up to almost 10^11^ phage mL^-1^ are seen for hibernation mode. This raises questions as to at what stage of phage development this pause takes place, what triggers the pause, and what the state of the bacterial cell is during the prolonged hibernation mode of T4 infection that allows it to remain functional and able to respond to nutrients with massive production of phage.

Alternatively, infected cells may display a “scavenger” response, where T4 uses whatever resources are available to gradually make a few progeny phage per cell over the course of 2 to 5 h after infection, but shows no response to later nutrient addition. The specific details of this scavenger response remain unclear and presumably vary depending on what is available, hence its categorization as a generalized ability to respond to the conditions it encounters, rather than as a specific mode of infection.

### Host DNA Breakdown in Stationary Phase

One important consequence of T4 infection of exponential-phase cells is gradual breakdown of the host DNA. This does not happen immediately after phage infection, although host transcription is shut off very rapidly. Rather, the release of mononucleotides largely parallels the production of progeny DNA (Kutter and Wiberg, 1968), while the residual host DNA is held bound in very large chunks to the membrane by many molecules of the small T4 ndd protein. The enzymes responsible for the degradation, which work in two stages, are made following delayed-early kinetics, and the released nucleotides flow directly into the phage-encoded nucleotide synthesizing complex (Kutter et al., 1994c). Their re-utilization for phage DNA synthesis is so efficient that host DNA breakdown can only be observed by using a phage mutant defective in phage DNA synthesis.

**FIGURE 3:**
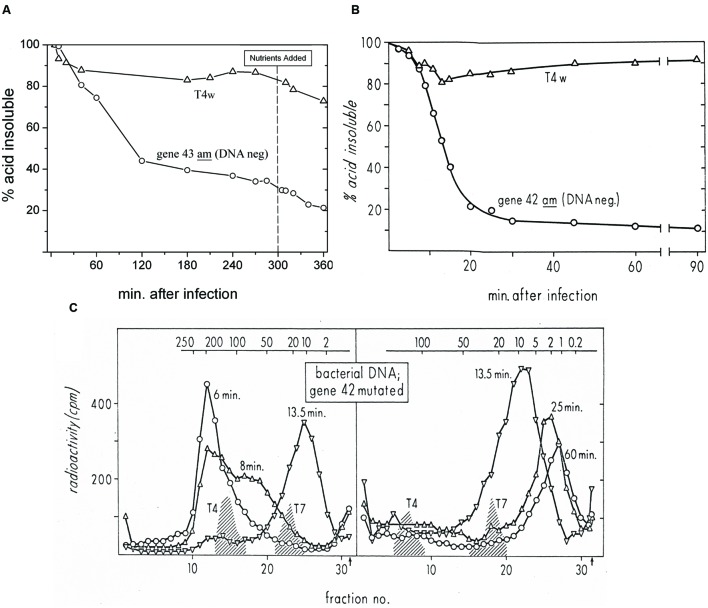
**(A)** Host DNA degradation analysis of ZK126 that was labeled with tritiated thymidine during exponential growth and then infected at 48 h in stationary phase with either T4D or T4 am4332 (DNA polymerase^-^), in parallel with infection of cultures being tracked in terms of phage production and BS. **(B)** Status of DNA labeled with tritiated thymidine over the course of similar infections of *E. coli* B with T4D and T4 amN55x5 (dCMP HMase^-^) in exponential phase, as reported by Kutter and Wiberg (1968). Both T4 am4332 and T4 amN55x5 produce DNA^-^ phenotype phage when grown on a non-amber suppressor strain such as *E. coli* B or ZK126. **(C)** Sucrose gradient analysis determining the size of the acid-insoluble fraction of the host DNA at various times after this exponential phase infection of *E. coli* B by T4D. “T4” and “T7” refer to phage DNAs used as sedimentation markers.

We have now carried out similar examinations of host DNA breakdown in 2-day-old stationary-phase cells in M9 supplemented with glucose and CAA, as above. For these studies, 4 mL was taken out of the culture as soon as it reached an OD of 0.4 on the first day and ^3^HdT as well as unlabeled dA and dT were added to this aliquot, as described in Section “Materials and Methods,” to label the bacterial DNA as it is being produced. At 48 h after the start of the culture, both the radioactive and non-radioactive cultures were split in two just before phage were added: either T4D or T4 DNA polymerase mutant am4332, at an MOI of about 10 (**Figure 3**).

In our various radiolabeling experiments, including the one presented here, we saw very little phage production by T4 prior to nutrient addition. Nutrients were added at 5 h post phage addition and at that point, we saw the strong nutrient response characteristic of a predominantly hibernation mode infection, yielding over 3 × 10^10^ phage per ml, while no phage production was observed before or after nutrient addition with am4332 (data not shown). As seen in **Figure 3A**, after wild-type infection there is only a small drop in the amount of radioactive label present in DNA, as was also reported for T4 exponential-phase infection of *E. coli* B by Kutter and Wiberg (1968); that data is reproduced here in **Figure 3B**. After infection of the same stationary-phase cells with T4 am4332, however, there was a 40% drop in acid-precipitable ^3^HdT by 60 min after infection, and by 120 min over half of the host DNA was in acid-soluble form. As seen, though degradation is substantially slower in stationary phase, the general patterns for both the wild-type T4 and the mutant phage unable to make DNA are quite similar to those of the 1968 work infecting exponentially growing *E. coli* B.

In the 1968 exponential-phase experiments, sucrose gradient analysis showed that the residual acid-insoluble DNA is being gradually cut into large pieces (reproduced here as **Figure 3C**) by a combination of cytosine-specific phage-encoded endonucleases II and IV, while the degradation from there to mononucleotides for reutilization was shown to require T4’s gene 46- and 47-encoded exonuclease, which is also involved in T4 recombination and thus not specific for cytosine DNA. In the absence of expression of genes 46 and 47, the sizes of the remaining fragments at various times after infection were the same as those seen here for T4, but all of the DNA was in the respective peaks rather than only the fraction indicated in **Figure 3B** as not yet having been degraded to mononucleotides.

Since the host DNA is so substantially degraded to an acid-soluble form after infection by the DNA polymerase mutant in stationary phase, we infer that it is similarly degraded in the parallel wild-type T4 infection, as was shown in the exponential phase sucrose gradient experiments, with the released host nucleotides being efficiently re-incorporated into phage DNA. The endoII and endoIV that initiate host DNA degradation are under middle-mode regulation, as are genes 46 and 47 and the many enzymes involved in nucleotide biosynthesis and DNA replication (Cowan et al., 1994).

**FIGURE 4:**
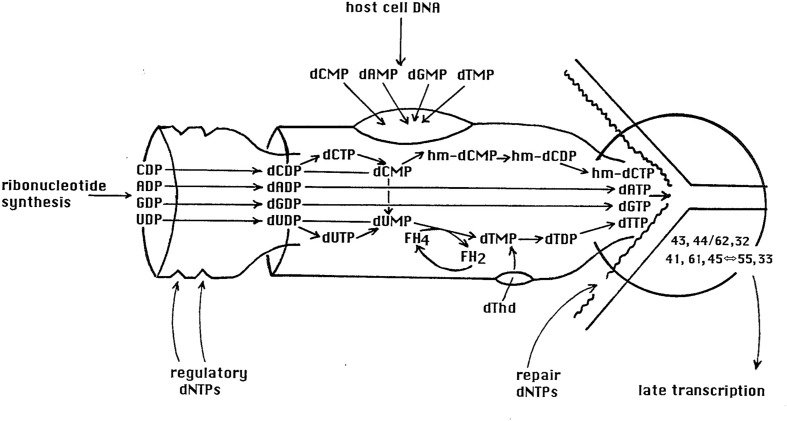
**The tight T4 nucleotide synthesizing complex, funneling NDP nucleotides very efficiently into the dNTP nucleotides needed for synthesizing T4 DNA, in precisely the 2:1 ratio of A and T to G and C required by T4.** The dNMP breakdown products of host DNA are also funneled through this complex, which feeds directly into T4’s DNA synthesizing complex. This unpublished diagram was designed by Chris Matthews at Oregon State University, who long led its study.

Additionally, since the host DNA is being degraded, it seems highly probable that the observed rapid response to added nutrients in hibernation mode is dependent on stable host proteins and/or stable host RNAs. It appears unlikely to be the result of a transcriptional response, particularly assuming that the host DNA in infected cells is broken down in two steps in stationary phase as it is in exponential phase (**Figure 3C**), making it likely that the residual acid-insoluble DNA in cells in hibernation mode is in relatively small fragments, as it is in exponential phase. Such fragments of DNA might be large enough to be transcribed, except for the fact that one of the first-made T4 proteins, gpAlc, blocks all transcription of cytosine-containing DNA (Kutter et al., 1981). The efficiency with which the ^3^HdTMP from host DNA degradation is reused suggests that the elaborate T4 nucleotide synthesizing complex (diagrammed in **Figure 4**) has already been produced by the time of the degradation to acid-soluble form, that it is capable of efficiently channeling the nucleotides from host DNA breakdown into the DNA replication fork as it does in exponential phase, and that enough DNA is already being synthesized to largely use those nucleotides. It thus appears most likely that the lack of phage production in hibernation mode is due to some sort of blockage of late-gene transcription and/or translation, which is quickly reversed once the glucose and CAA again become available.

### Ability of *E. coli* Infected with T4 well into Stationary Phase to form Infective Centers

All of the above stationary-phase experiments were conducted at 48 h after inoculation of the bacteria into fresh medium, leaving the question of how late into stationary phase T4 can still infect *E. coli.* A simple assessment of that question was carried out by exploring the ability of T4 to form infective centers (visible as plaques) when added at very low MOI to a culture at up to 19 days after inoculation into the same medium used for the above experiments.

**FIGURE 5:**
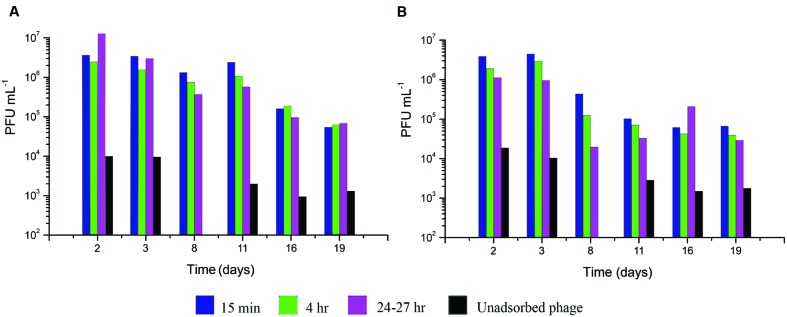
**Infective center production and stability after low MOI T4 infection at various lengths of time after *E. coli* inoculation into M9 supplemented with CAA and glucose.** At each time point, 1 × 10^7^ phage/mL were added: **(A)** in ZK126 and **(B)** in ZK1000. The number of infective centers and their stability was measured at 15 min, 4 and 24 h by serially diluting the cells and plating 0.1 ml of the appropriate dilutions on ZK126, as previously described.

T4 infection of *E. coli* in exponential phase is highly efficient; when phage are added to exponential phase *E. coli* at an MOI<<1, diluted as necessary and plated, the number of infective centers observed is equal to the number of phage that were added to that sample, as determined by other methods. This is, in fact, the basis for the way phages like T4 are normally counted. To determine how well the ability to form infective centers is preserved as the bacteria progress further into stationary phase, 10^7^ phage per mL (MOI ~0.01) were added to stationary phase *E. coli* ZK126 and ZK1000 at various times up to 19 days after inoculation. At each time, samples were taken at 15 min, 4 and 24–27 h after infection, diluted on ice through unsupplemented M9, and immediately plated using TSA plates, where ample nutrients are available (**Figure 5**). To determine the level of unadsorbed phage, a sample was also taken at 10 min after infection into phage buffer with several drops added chloroform, vortexed well, and allowed to sit for at least 20 min before further dilution in phage buffer and plating on the same host. This lyses the infected cells before they would have completed making any progeny phage, so only the unadsorbed phage can form plaques, allowing determination of the actual numbers of infected cells that are producing infective centers.

At all times tested, at least 99.9% of the phage were able to adsorb. Interestingly, even better adsorption was observed after 11 days of growth for both host strains. For at least the first 3 days, over half of the infected cells were able to make infective centers, which were stable for at least 24 h, regardless of whether or not the host had a functioning stationary-phase sigma factor. On ZK126, 10–20% of the added phages formed infective centers even after 8–11 days, and a large fraction of those were stable for over 24 h. By 19 days, only about 1% still had found hosts where they could form infective centers; those were also still stable for 24 h.

For ZK1000, where the stationary-phase sigma factor gene has been deleted, the level of binding was similar to that seen with ZK126, but only about 5% of the cells infected at 8 days after inoculation could make infective centers, and this dropped to 1% for cells infected at 11 days and beyond. These results, coupled with the greater susceptibility to early lysis at higher MOIs (**Figures 2C,D**), indicate that while σ^S^ status may not be much involved in the regulation of T4’s infection patterns, it does play a role in providing a more stable host for infection under longer term and more stressful conditions.

## Discussion

T4 infection of exponentially growing *E. coli* quickly disrupts host genome structure and expression, largely by making use of T4’s complete substitution of HMdC for dC in its DNA (Kutter et al., 1994c). Elongation of transcription of all cytosine-containing DNA is terminated and host DNA replication is disrupted. The host DNA is gradually degraded, very efficiently funneling the released nucleotides into T4’s elaborate nucleotide synthesizing complex, as discussed above (**Figure 4**). In addition, translation of host mRNA is very rapidly and universally terminated (Wiberg and Karam, 1983); the mechanism for this has still not been determined, but very little of the *lac* mRNA transcribed before or just after T4 infection is bound to ribosomes, whereas virtually all *lac* mRNA in uninfected cells is ribosome associated (Kennell, 1970). At least eight new proteins, ranging from less than 10 kilodaltons (KD) to about 48 KD, are found bound to the ribosome following infection, but none of these proteins have yet been identified genetically. T4 infection also rapidly makes *E. coli* resistant to streptomycin because the ribosomes can no longer bind the antibiotic (Wiberg and Karam, 1983).

Little is known about the effects of these various mechanisms when T4 encounters cells which are in stationary phase and which therefore have made drastic metabolic and structural adaptations to the lack of cell growth. The data presented here indicate that there are at least two possible outcomes when T4 infects stationary phase cells.

Firstly, results presented here indicate that T4 has the option of responding to the starvation state of the cell by entering what, we call a “hibernation” mode, a reversible persistent dormant state in which T4 initiates phage protein synthesis but then halts phage development midstream. Once needed nutrients become available, phage development resumes and the standard lytic infection process continues to completion, producing large numbers of progeny phage. This sort of “hibernation” mode would protect the phage from environmental factors, giving the phage a safe haven for a prolonged period of time, and would also give it a competitive advantage against unbound phage when nutrient conditions abruptly change to allow bacterial growth. Interactions somewhat similar to this have sometimes been referred to as a “pseudolysogenic” state (Łoś et al., 2003; Abedon, 2009; Łoś and Wegrzyn, 2012). However, the term “pseudolysogeny” is used to describe a number of very different phenomena in the literature (Abedon, 2009; Hobbs and Abedon, 2016) so, we choose to avoid using the term here. This state is not to be considered a potential path to lysogeny, since the bacterial DNA is substantially degraded, making it is very clear that the host can never go on to replicate.

Secondly, it appears that T4 can also engage in what we call a “scavenger” response that takes advantage of whatever few nutrients are present, including nutrients released by any cells lysed from without or within, to produce small quantities of progeny. In this case, the cycle appears to run to completion within a few hours, with no ability to make further phage if nutrients later become available. This may be similar to T7’s behavior during infection of stationary phase cells, leading to its reported ability to produce a six orders of magnitude increase in phage titer while transiting the axenic mouse gut, where the level of T4 increased only 300-fold – i.e., the size of one burst in exponential phase (Weiss et al., 2009). T7’s infection of stationary-phase cells on plates – allowing the plaques to grow ever larger on the plates as each infected cell releases enough phage to continue plaque enlargement even once the lawn is well into stationary phase – is probably related to that finding; a similar phenomenon is observed for some small, tailless coliphages such as phiX174.

What regulates the choice between hibernation mode and the scavenger response is unclear, but it appears to be determined on a cell-by-cell basis and not to be specifically linked to host σ^s^ expression. We suggest that hibernation mode is the phage production state that is actively regulated, with the scavenger response representing any possible phage production using whatever resources are available if hibernation mode is not triggered. The detection of host DNA breakdown and nucleotide reincorporation into phage DNA within the first few hours after phage infection is indicative that there is indeed middle-mode protein synthesis even before nutrient addition. However, we see no sign of an increase in completed phage particles in hibernation mode until after the addition of nutrients. All of our evidence to date suggests that it is late-mode transcription (or possibly translation) which is delayed by an unknown mechanism until adequate resources become available. We know that the infection does not progress to the production of phage and completion of the lytic cycle until after nutrient addition, but we cannot yet identify the precise point at which hibernation mode suspends phage production.

In stationary phase, we know that at least enough DNA is produced to incorporate most of the nucleotides from the host DNA. We do not yet know if progress is blocked at the level of late-gene transcription or translation or later. A very unusual sigma factor, gp55, is essential for T4 late-gene transcription (Williams et al., 1994). It recognizes a promoter that has no -35 region; it only interacts with a -10 stretch of nucleotides in the usual promoter region. However, gp55 also interacts directly through gp45 with the DNA synthesizing complex which is depicted in **Figure 4**, coupling the level of late mRNA and thus late protein synthesis to a substantial degree to the production of DNA to be packaged into the procapsids (Geiduschek and Kassavetis, 2010). During exponential phase infections, the T4 DNA presents as a long multi-branched concatemer, containing approximately 50 T4 equivalents of DNA. The DNA is packaged into the heads from the ends of the branches by a head-full mechanism, with any nicks being repaired and side branches clipped off in the process by the packaging enzymes. Since the control of late transcription is specifically linked to DNA replication at least in exponential phase, this suggests that at least some late-mode transcription may well have already occurred, and the possibility that hibernation mode control of the phage program happens at the translational level should be considered. For example, one factor which could conceivably play a role in the rapidity of the response to new nutrients is the sudden increase in functional ribosome numbers as the ribosomal dimerization characteristic of stationary-phase cells is virtually instantaneously reversed (Shimada et al., 2013).

Stationary-phase changes in the bacterial surface could also interfere with the infection process, making some phages unable either to adsorb properly or to transfer their DNA into the potential host’s cytoplasm, but we see no indication of binding problems for the interaction of T4 (or coliphage T5, data not shown) with the tested lab strains of *E. coli* in stationary phase. We did, however, find that some other coliphages in our collection were far less capable of binding to stationary phase ZK126 and ZK1000 and showed no phage growth either before or after nutrient addition (data not shown).

### Integration of these Observations with more Ecological Studies of T4 and Related Phages

The ability of any given phage to successfully infect stationary phase cells is likely to be influenced by its natural hosts and habitat, population reservoirs and life cycle.

Various lines of evidence indicate that T4 is specifically adapted to the colonic environment of mammals and has developed strategies to optimize survival in those conditions. For example, T4 requires specific monovalent cation concentrations most commonly found in colonic environments to efficiently bind to *E. coli*. It is incapable of binding in fresh water, and binding efficiency was greatly reduced in the high salt concentrations commonly found in sea water (Abedon, 1990). Furthermore, T4B also needs tryptophan – a nutrient not commonly found in extra-colonic environments, but, present in fecal water – to successfully bind, though T4D has no such known requirements. Furthermore, T4’s optimum temperature for infection is about 37–42°C, above which phage production drops off precipitously, while the intracellular osmolarity has surprisingly little effect over a range from 58 to 630 mOsm, despite its effects on phage assembly *in vitro* (Kutter et al., 1994a).

In the ever-changing colonic environment, the ability to either produce low levels of phage as quickly as possible or to so effectively ‘reserve’ a host for greater future progeny production would give T4 a competitive advantage against other coliphages. A clinical study in Bangladesh showed that some of the T4-related phages orally administered to diarrheal patients experienced elevated phage titers in stool samples, suggesting that intestinal phage amplification can occur (Bourdin et al., 2013), though little or no increase was observed in Nestle’s Bangladesh infant diarrheal clinical trials, where lower than expected levels of *E. coli* were found in the stools (Sarker et al., 2016). Scavenger response growth would maintain a small population of free T4 in the colonic environment. Hibernation mode, in contrast, would give T4 a longer-term survival mechanism as well as a temporal advantage when more nutrients become available, since hibernation mode infected cells start producing phage almost immediately after re-addition of usable nutrients, as opposed to having to wait until their hosts have started growing to begin the infection process. This would be consistent with the broad range of adaptive strategies known for T4 and related phages. For example, T4 has evolved a mechanism that allows infected cells to detect related extracellular phages attempting to super-infect the cell and delay the lysis of phage-pregnant cells to release progeny for about 6 h, giving T4 an advantage where there are substantially more phage around than susceptible hosts (Paddison et al., 1998).

## Conclusion

T4 is able to enter and take over stationary-phase cells, finding a longer-term safe haven in at least some of the cells it infects in stationary phase. In this “hibernation” mode, the phage production process pauses at some point after DNA synthesis is in progress – but before the completion of the T4 capsid – until new nutrients become available. It then produces substantial numbers of phage in short order – without having to wait through the usual lag phase before uninfected cells would start multiplying, and much faster than it would start releasing phage in exponential phase. T4 is also capable, after exposure to stationary phase cells, of producing progeny phage over a period of 2–5 h, a rate far slower than that seen in cells growing exponentially. This “scavenger” response infection renders the cell incapable of responding to fresh nutrients, and some of the cells appear to lyse very early without making progeny, especially in *E. coli* lacking the stationary-phase sigma factor. This early lysis may provide additional nutrients to any intact infected cells, allowing for a small amount of phage production without additional nutrient addition.

Further work is planned to explore what if any T4 genes are involved in hibernation mode, whether other T4-related phages share this extra capability, and how widely it has evolved within that subfamily and beyond – particularly whether it is found in the RB49/MEV12 group of phages, which do not have HMdC in their DNA and thus are more limited in their tools for taking over the host.

## Author Contributions

Primary responsibility for conception and design of the work: EK. Substantial contributions to the design of the work and acquisition, analysis and interpretation of the data: DB and AE-S. Substantial contribution to the acquisition, analysis and interpretation of the data: EK, ZH, and JP. Drafting the work and revising it critically for important intellectual content: EK, DB, and AE-S. Revising the work critically for important intellectual content: ZH and JP. Final approval of the version to be published AND agreement to be accountable for all aspects of the work in ensuring questions related to the accuracy or integrity of any part are appropriately investigated and resolved: EK, DB, AE-S, ZH, and JP.

## Conflict of Interest Statement

The authors declare that the research was conducted in the absence of any commercial or financial relationships that could be construed as a potential conflict of interest.
