# Supplementary figures and images for: Bacteriophage T4 Infection of Stationary Phase E. coli: Life after Log from a Phage Perspective
Source: Front Microbiol. 2016 Sep 8;7:1391. doi: 10.3389/fmicb.2016.01391 (PMC5014867; doi:10.3389/fmicb.2016.01391)

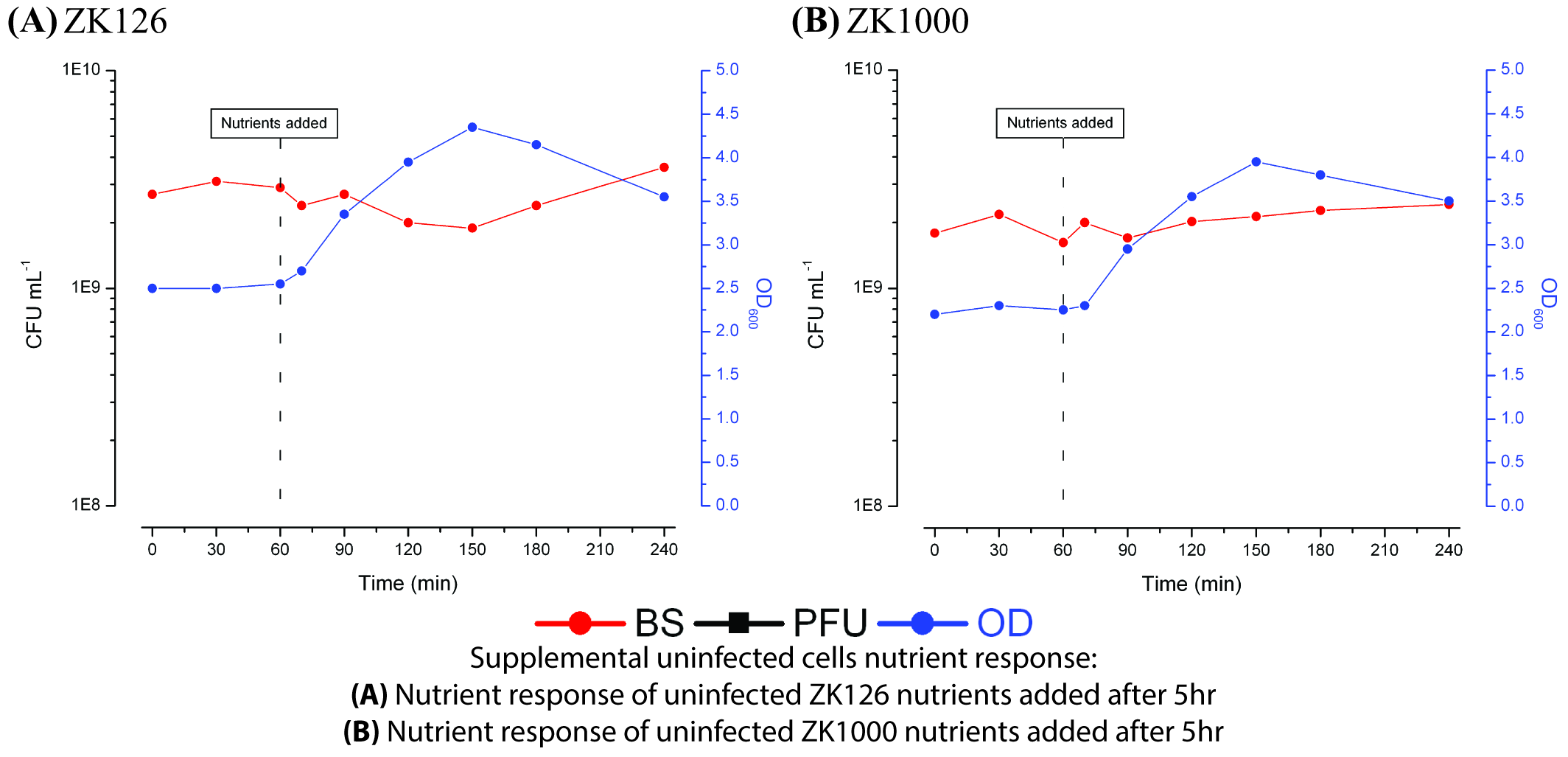

Supplement: Supplementary file 1 [file Image_1.TIF]

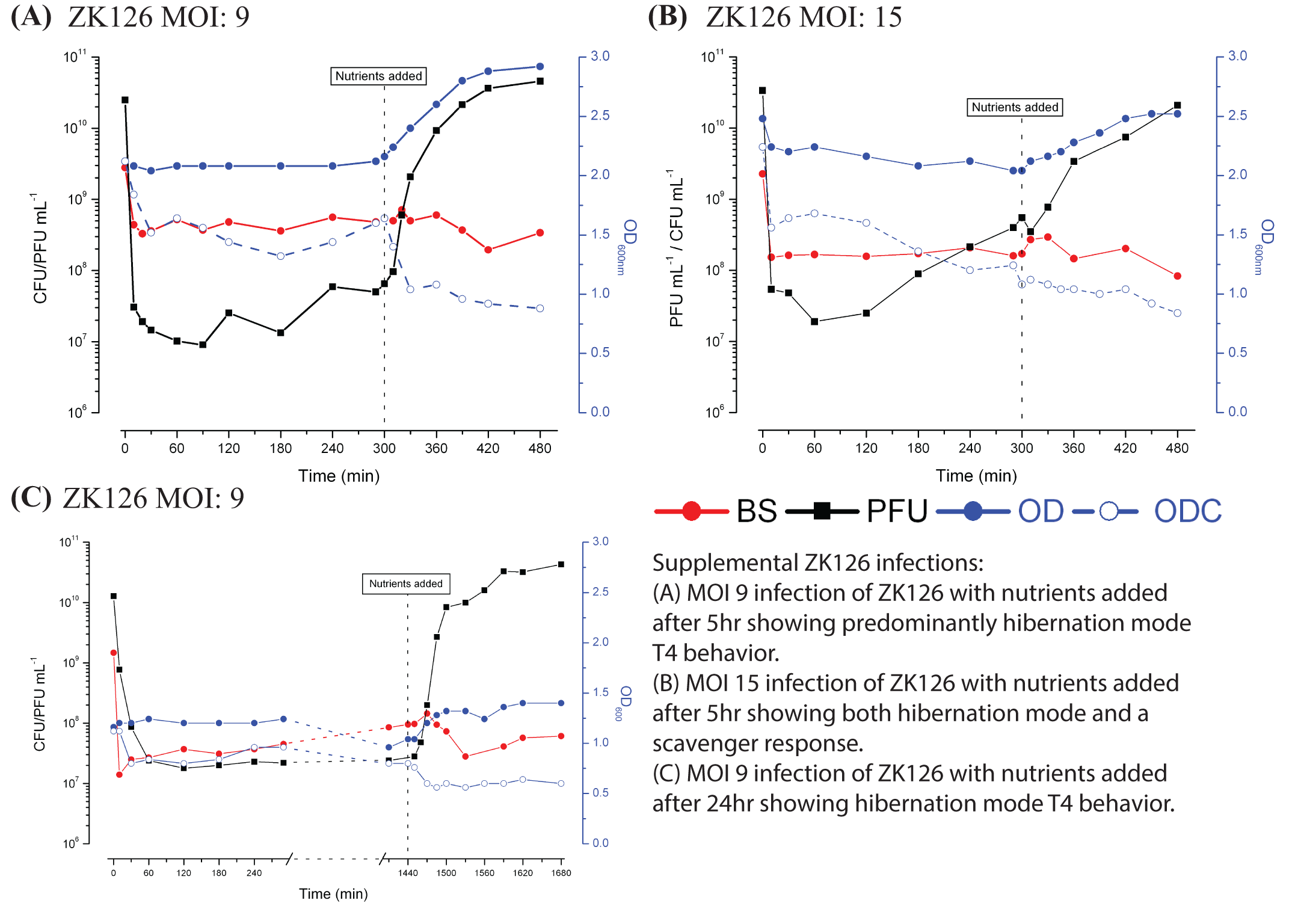

Supplement: Supplementary file 2 [file Image_2.TIF]

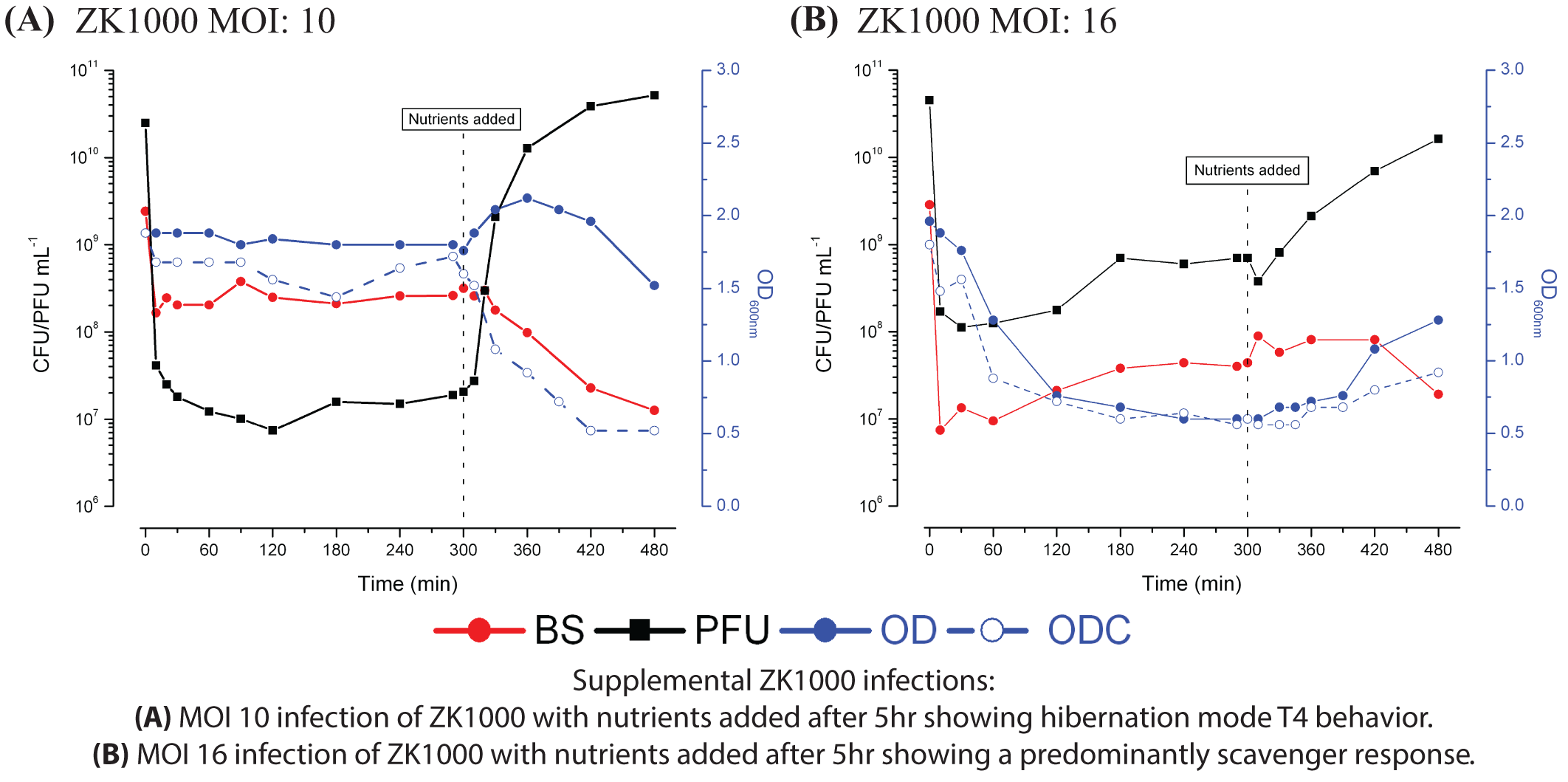

Supplement: Supplementary file 3 [file Image_3.TIF]

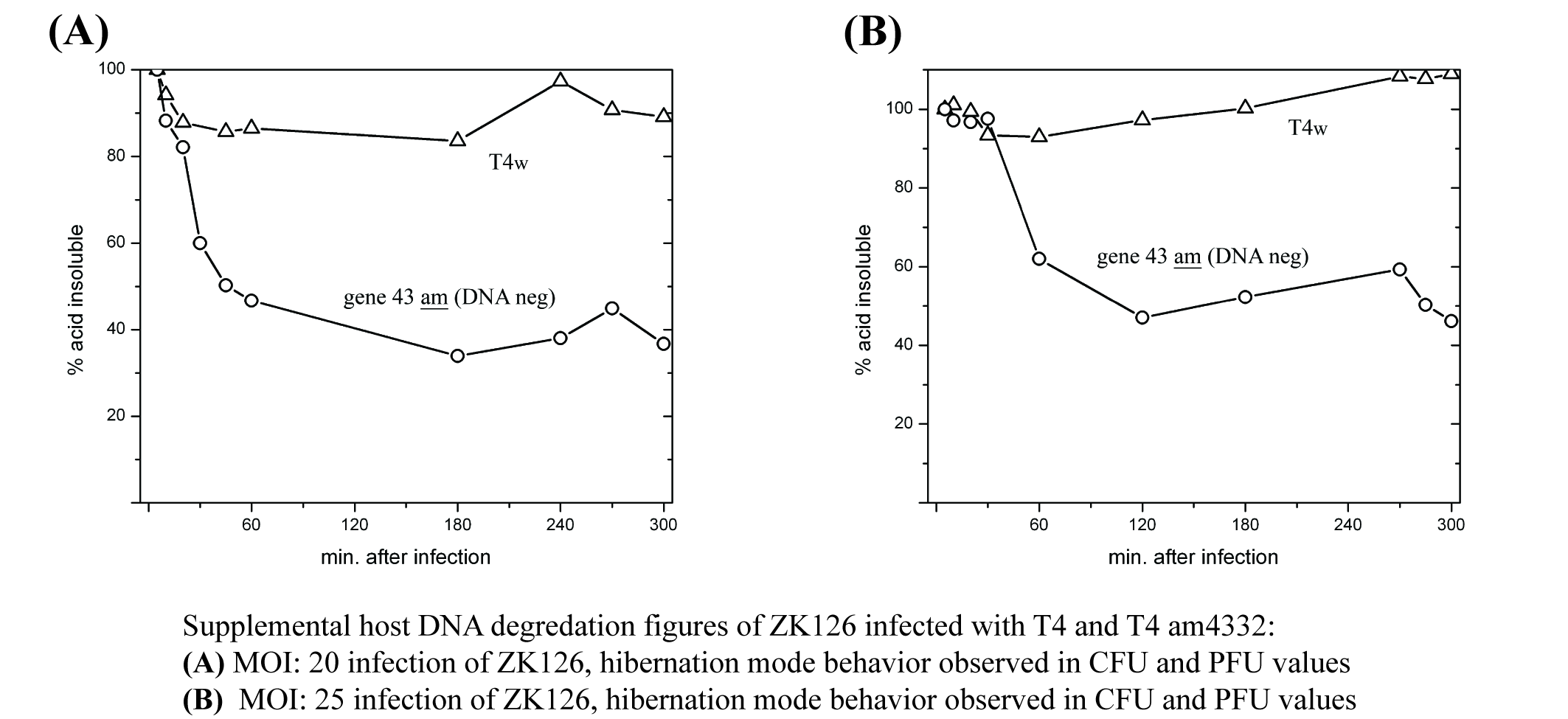

Supplement: Supplementary file 4 [file Image_4.TIF]
